# Analysis of the emotional status of healthcare services in the context of the Covid 19 pandemic

**DOI:** 10.1192/j.eurpsy.2023.883

**Published:** 2023-07-19

**Authors:** M. Kubiak, M. Waszczak-Jeka, S. Jeka, P. Żuchowski, E. Mojs

**Affiliations:** 1 Department of Clinical Psychology, Poznan; 2 Clinical Medicine, Warsaw; 3 University Hospital no 2; 4 Collegium Medicum, Bydgoszcz, Poland

## Abstract

**Introduction:**

The psychological burden of the COVID-19 pandemic can have a lasting impact on the emotional well-being of healthcare workers (HCWs). Healthcare professionals working during the pandemic experienced increased occupational stress due to the high risk of contracting the virus, work overload, significantly increased working hours and overlapping job responsibilities

**Objectives:**

The study aimed to assess the dynamics of mental health and coping and to analyze how HCWs’ emotional responses to a pandemic at the beginning of the pandemic and at the two-year follow-up. This study compared the results of examination of emotional response of HCWs in 2020 and 2022. The relationships between stress and alexithymia, emotional processing, and negative / positive affect in healthcare professionals were analyzed.

**Methods:**

All respondents of the study were hospital or ambulatory healthcare professionals. Overall, 285 subjects were examined in 2020 while 252 subjects were examined in 2022. Respondents completed several questionnaires such as e.g., the Toronto Alexithymia Scale-20, the Perceived Stress Scale (PSS-10), the Emotional Processing Scale (EPS), and the Positive and Negative Affect Scale (PANAS).

**Results:**

Significant increased scores were observed forthe following parameters: alexithymia, identifying feelings, verbalizing feelings, operative thinking style, emotional processing, suppression, signs of unprocessed emotion, controllability of emotions, avoidance of emotional triggers, impoverished emotional experience, surrogate activities, denial, substance use, self-blame, stress, negative mood, somatic disorders, concern, dysfunction, depression, anxiety state and trait.

The results were significantly lower for planning, using instrumental support, positive reframing, acceptance, religion, using emotional support, venting and positive mood.

Statistically significant differences could not be found for acceptance, active coping, humor and behavioral disengagement

**Conclusions:**

Research indicates that exposure to the pandemic a pandemic is associated with greater severity of a range of symptoms in HCWs. Two years of the pandemic may have led to psychological impairment. HCWs are more likely to use coping focused on emotional freezing, suppression, avoidance and / or an increase in negative emotions. Clinical observations indicate that caring for others does not correlate with appropriate self-care

**Disclosure of Interest:**

None Declared

